# Distance to vaccine sites is tied to decreased COVID-19 vaccine uptake

**DOI:** 10.1093/pnasnexus/pgad411

**Published:** 2023-12-06

**Authors:** Asaf Mazar, Daniel Jaro, Geoff Tomaino, Ziv Carmon, Wendy Wood

**Affiliations:** The Wharton School, University of Pennsylvania, Philadelphia, PA 19104, USA; Center for Psychedelic & Consciousness Research, Johns Hopkins University, Baltimore, MD 21218, USA; Department of Marketing, Warrington College of Business, University of Florida, Gainesville, FL 32611, USA; Department of Marketing, INSEAD, Singapore 138676, Singapore; Department of Psychology and Marshall School of Business, University of Southern California, Los Angeles, CA 90089, USA

**Keywords:** vaccination, friction, vaccine hesitancy

## Abstract

COVID-19 remains a leading cause of mortality in the United States, despite the widespread availability of vaccines. Conventional wisdom ties failure to vaccinate primarily to vaccine-skeptic beliefs (e.g. conspiracy theories, partisanship). Yet in this research, we find that vaccination is also hindered by travel distance to vaccine sites (a form of *friction*, or structural barriers). In study 1, Californians living farther from vaccine sites had lower vaccination rates, and this effect held regardless of partisanship. In study 2, Chicago zip codes saw an uptick in vaccination following vaccine site opening. These results proved robust in multiverse analyses accounting for a wide range of covariates, outcomes, and distance indicators. COVID-19 vaccination is hampered not only by vaccine hesitancy but also by structural barriers like distance. Efforts to boost vaccination could benefit from minimizing friction.

**Significance Statement**

Vaccination offers potentially life-saving protection against COVID-19. And yet, many in the US remain unvaccinated. This research shows that friction–specifically, distance to vaccine sites–is a pivotal barrier to vaccine uptake. Using geospatial methods, we find that longer distances to vaccine sites are tied to reduced vaccine uptake (Study 1) and that vaccination in an area increases once a nearby vaccine site opens (Study 2). Multiverse analyses demonstrated that the observed distance effects are robust to a wide range of alternative explanations and model specifications. These findings illustrate how minor friction can curtail even a one-time action with potentially life-saving benefits, and highlight the promise of policies that promote vaccination by reducing friction.

## Introduction

Despite the broad availability of COVID-19 vaccines, undervaccination remains a challenge (1), with COVID-19 still a leading cause of death (2). What hinders vaccine uptake? This question is key to addressing the near-certain emergence of future pandemics (3).

Vaccine-related beliefs—such as concerns about vaccine side effects, and political partisanship—have attracted considerable attention (4). Although beliefs matter (5), the focus on beliefs often overshadows friction—external barriers like distance to vaccine sites (6). In illustration, among New York Times vaccine-related op-eds published 2021–2022, at least 73 dealt with misinformation, compared with only 4 addressing allocation of resources (the category most closely related to friction; see SI Appendix). It is perhaps no surprise that the media overlooked travel distance, given that people in the United States tend to discount friction (6). In two pilot surveys (*n* = 199 and 361), we found that Americans overwhelmingly ignored distance: When listing barriers to COVID-19 vaccination, a few participants—6 and 3%, respectively—mentioned distance at all (see SI Appendix).

Yet living far from health care is tied to worse health outcomes (7).^a^ This pattern may hold for COVID-19 vaccination as well, since some Americans live where vaccine accessibility is low (9). The present work builds on this prior research by testing whether longer distances to vaccine sites are tied to undervaccination. If so, vaccination policies should not only target beliefs but also minimize friction (10).

All data, code, and materials are available online: https://osf.io/brmy4/?view_only=eb9624efbaa04fd396a9a6084ba4a21e. Detailed information on data sources are given in the SI Appendix.

## Study 1

In study 1, we leveraged California's granular COVID-19 geodata to test whether longer distances were tied to lower vaccine uptake among all California zip codes. We quantified distance using both geographic distance (using vaccine sites’ geolocation) and travel time (leveraging Google Maps’ API). We employed a multiverse approach (11), fitting a comprehensive set of theoretically justifiable models to test for robustness across a wide range of vaccination outcomes, distance indicators, variable transformations, and covariates.

**Table 1.** Model specifications used in study 1 multiverse analyses.

Vaccine uptake outcome	% partially or fully vaccinated % fully vaccinated % who received a booster dose
Distance measure	1–4. Number of vaccine sites within a [1/5/10/20] mile aerial radius 5–8. [Median/mean] [travel distance/travel time] between each zip code and the 10 nearest vaccine sites
Outcome transformation	None Log
Covariates	None Population density Share of seniors Median household income Urban/rural designation Share of Trump vote in 2020 Share of non-Hispanic white Vaccine hesitancy (county level; from the US Census Bureau Household Pulse survey)

Travel distance and time were measured between vaccine sites and each zip code's population-weighted centroid, as defined by the US Department of Housing and Urban Development. All covariates were measured on the zip-code level, except for vaccine hesitancy, which was available only on a county-level basis.

### Results

The analysis included 1,454 zip codes and 3,531 vaccine sites. We computed 6,096 multilevel regressions (with zip codes nested within counties) predicting vaccine uptake using every possible combination of: (i) 3 vaccine uptake measures, (ii) 16 distance measures, and (iii) up to 7 covariates: vaccine hesitancy, share of 2020 Trump votes, population density, urban/rural status, household income, share of non-Hispanic whites, and share of seniors (i.e. we fit models with every possible combination of one covariate, two covariates, etc.; see Table 1).

**Table 2.** Model specifications used in study 2 multiverse analyses.

Vaccine uptake outcome	Overall vaccination count (65 and over)^a^ First vaccination count (65 and over) Overall vaccination count (all ages) First vaccination count (all ages)
Outcome transformation	None Log
Existing vaccine sites within a 1-mile radius^b^	All zip codes (no subsetting) Zip codes with less than eight existing sites Zip codes with less than four existing sites
Covariates	None Citywide vaccination rate Population density in zip code Percent seniors in zip code Median household income in zip code Percent non-Hispanic white in zip code

^a^Subset used because during most of the study period, only seniors were eligible for vaccination.

^b^Number of existing vaccine sites prior to the new vaccine clinic's opening. Four and eight vaccine sites represent the 33rd and 66th percentiles of the distribution of existing vaccine sites, respectively.

**Fig. 1.**
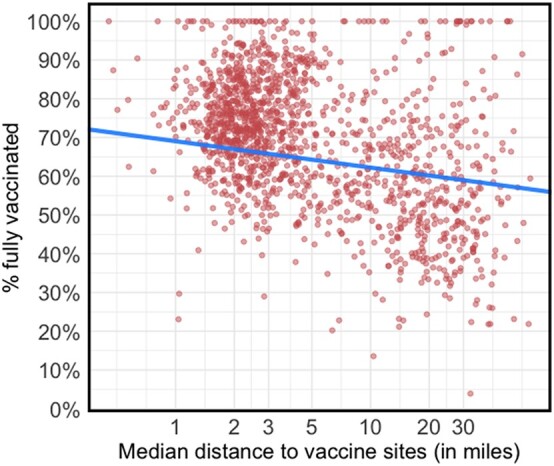
Illustration of a multilevel model predicting vaccination from distance. Multilevel model predicting fully vaccinated share of the population as a function of log median distance to vaccine sites. Similar results emerge when using as an outcome as the share of the population with at least one dose or who received a booster dose.

Supporting a unique role of friction, a sizable association between distance and vaccination emerged, even when controlling for covariates and spatial clustering (see Fig. 1 for an example illustrating one model). Supporting the link between increased distance and undervaccination, the association between distance and vaccine uptake was negative in 5,955 models (98%) and negative and significant in 5,202 models (86%).

We used Simonsohn et al.'s (12) procedure to assess whether the share of significant negative effects in the multiverse was significantly higher than would be expected under the null. Using 500 bootstrapped samples, we found that the proportion of negative and significant effects, 0.86, was higher than the 99.95% percentile of null samples, 0.70, i.e. the results are significant with *P* < 0.001.

Distance hampered vaccine uptake regardless of partisanship. That is, in a separate multiverse analysis, the interaction between distance and share of Trump votes was nonsignificant at the 0.05 level (62% nonsignificant effects, 31% significant and negative, and 7% significant and positive; see SI Appendix).

## Study 2

Study 1 revealed that among all California zip codes, longer distances to vaccine sites were tied to reduced vaccine uptake. To probe this in a longitudinal setting, we used vaccine site opening data from Chicago, IL. Vaccine site opening poses a natural quasi-experiment testing friction effects, in that vaccination rates should rise in an area once a nearby site opens. By tracking vaccine uptake within a zip code before and after vaccine site opening, we mitigate confounding factors that could cause some zip codes to have better vaccine site accessibility. We again used a multiverse approach to test the effects’ robustness across a range of analytic choices.

### Results

We used the data provided by Chicago because it includes both zip-code level vaccine uptake and vaccine sites’ opening dates. The data included 48 zip codes and spanned 6,804 days, with most data points dating early in the vaccine rollout period, between January and April 2021. We computed 768 multilevel linear regression models (days nested within zip codes) comparing zip codes’ vaccine uptake 1 month before and 1 month after vaccine site opening from every possible combination of 4 vaccine outcomes, 2 outcome transformations, 3 subsetting thresholds, and up to 6 covariates (see Table 2).

Vaccine uptake significantly increased after vaccine site opening in 560 out of the 768 models (73%). Using the bootstrap procedure described earlier to assess statistical significance, we found that the share of significant positive results, 0.78, was significant at *P* < 0.001.

To further probe the multiverse analysis, the analyses were partitioned into models that predicted vaccination among seniors only (384 models, 50%) and among all age groups (384 models, 50%). Note that only seniors were eligible for the vaccine during most of the examined period. Consequently, overall vaccination rates include those who managed to receive a vaccine before becoming eligible—presumably a highly motivated subpopulation willing to travel far for a vaccine. Thus, vaccination among seniors presents a more valid test of our prediction in this dataset. Indeed, the effect differed substantially across the 2 subsets: When predicting vaccination among seniors, 384 (100%) model estimates were positive, and 368 (96%) were positive and significant. Among the overall population, 192 (50%) models predicting vaccination were positive and significant (37.5% were nonsignificant, and 12.5% were negative and significant).^b^

## Discussion

Distance is a potent barrier to vaccine uptake. Distance effects proved remarkably robust, holding across a wide range of measures and after accounting for potent covariates such as vaccine hesitancy and partisanship. Further, distance effects held regardless of partisanship. These findings highlight friction as a key force shaping behavior—one that is often absent from public discourse.

Reducing distance may be a promising, noncoercive lever for addressing undervaccination—one that does not require changing people's beliefs. Minimizing distance may thus be particularly effective for highly polarized issues such as COVID-19 vaccination in the United States. The United States has already taken some significant steps in this direction. For instance, initial vaccine rollout was often concentrated in a smaller number of vaccine supercenters. Although mass vaccination sites may be efficient (13), subsequent deployment integrated better into communities via local pharmacies. Still, this approach may fail those living in pharmacy deserts (14), which might call for different approaches, like working with community organizations (15).

Notably, the locations examined here—California and Chicago, IL—invested resources to promote equitable vaccine distribution. Distance effects may be even stronger in places without such measures. Although the present investigation concerned just COVID-19 vaccines, reducing friction can be a key to the delivery of other public health resources such as healthy food, exercise opportunities, and mental health care.

## Supplementary Material

pgad411_Supplementary_Data

## Data Availability

All data, code, and supplementary information for both studies are available online via the Open Science Framework: https://osf.io/brmy4/?view_only=eb9624efbaa04fd396a9a6084ba4a21e.

## Notes

a Indeed, related research finds that interventions that reduce logistical barriers to vaccination—like setting default vaccine appointments—can boost vaccine uptake (8).

b Note that zip-code level 2020 election voting data were not available for Chicago, and so we were not able to use this as a covariate as in study 1.

## Preprints

This manuscript was posted as a preprint: https://psyarxiv.com/mux5s/
