# Supplementary material for: Distance to vaccine sites is tied to decreased COVID-19 vaccine uptake
Source: PNAS Nexus. 2023 Dec 6;2(12):pgad411. doi: 10.1093/pnasnexus/pgad411 (PMC10718641; doi:10.1093/pnasnexus/pgad411)
Supplement: pgad411_Supplementary_Data [file pgad411_supplementary_data.docx]

# Supplementary Information

# Distance to Vaccine Sites is Tied to Decreased COVID-19 Vaccine Uptake

## Pilot Survey 1 Methods

## Participants (n = 199; 46.2% women; *M*_Age_ = 29.47, *SD*_Age_ = 10.19) responded to the following item: “In your view, what are the major influences towards somebody not getting a COVID-19 vaccine? You may list up to 10 influences.” A research assistant blind to the hypothesis then coded whether or not each of these influences represents distance to the nearest vaccination center (0 = not distance, 1 = distance).

## Pilot Survey 2 Methods

Pilot Survey 2 used a between subjects design. We asked participants (n = 361; 49.3% women; *M*_Age_ = 39.76, *SD*_Age_ = 13.37) “In your view, what is a major influence on whether a (Democrat / Republican / person) would get vaccinated for COVID-19?” We then asked them whether they could think of another major influence. If so, after listing this influence, we again asked them if they could think of another. We repeated this process until each participant listed up to five influences. A research assistant blind to the hypothesis then coded whether or not each of these influences represents distance to the nearest vaccination center. The proportion of participants mentioning distance was largely equivalent across the 3 categories (Democrat / Republican / Person), and so was averaged.

##

## Study 1 Additional Multiverse Analyses - Interaction between Distance and Partisanship

## We ran this additional multiverse analysis using all models in the main multiverse analysis that included the share of 2020 Trump vote as a covariate (3,072 models total), with the addition of an interaction term between the relevant distance indicator and share of 2020 Trump vote. Thus, all models included a term for the main effect of distance, a term for the main effect of partisanship, and the interaction between them. Both distance and partisanship variables were centered to reduce multicollinearity. We then used the same bootstrap analysis used in the main multiverse analysis to calculate the interaction effect’s statistical significance.

## New York Times Op-Ed Analyses

We used the New York Times (NYT) Article Search API to assess how often NYT op-eds mentioned beliefs and friction. We queried all NYT op-eds that were published in the years 2021-2022 and included the term “vaccine,” retrieving 1,037 articles. We then examined keywords—standard tags that are associated with each story—and computed their frequency in the resulting dataset.

##

##

##

## Datasets

## Study 1

Our analyses used the following data sources.

**COVID-19 Vaccination Rates** Our main outcome was COVID-19 vaccination data provided by the California Health and Human Services Agency, as of November 22, 2022.^1^ This dataset provides weekly zip-code level vaccination rates for the state of California. Our analysis excluded zip codes that do not represent geographical units. Namely, 182 zip codes classified as a PO Box and 19 zip codes classified as “unique” (these are usually zip codes that represent a central mailing location for large organizations). To measure the distance between a given zip code and a vaccine site, we measured distance between a vaccine sites and each zip code’s population-weighted centroid, as defined by the U.S. Department of Housing and Urban Development.

**Vaccine Site Location** GISCorps’ COVID-19 vaccination provider geolocation data^2^ is a crowdsourced dataset created in collaboration with leading health providers (CVS Health, Walgreens) as well as state and local authorities. It includes exact geolocation (longitude and latitude) for 37,890 vaccination sites across the United States (3,531 vaccination sites in California).

**Vaccine Hesitancy** Vaccine hesitancy, using the Health and Human Services’ estimates, is derived from the Census Bureau’s Household Pulse Survey, which measures willingness to get vaccinated for COVID-19.^3^

**Voting Patterns** Precinct-level election results from California are hosted on the Harvard Dataverse.^4^ Precinct-level results were converted to the census-block level using the state of California’s General Election Geographic Data,^5^ aggregated to the census tract level and then converted to zip code using HUD-USPS crosswalk files (see “Data processing” below).^6^

**Population density, urban/rural status, median household income, share of seniors, and share of non-Hispanic white population** We used zip-code level census data for population density (number of residents per square mile), urban/rural status, median family income, and share of seniors, obtained using the “zipcodeR” package. The share of the non-Hispanic white population was taken from the 5-year 2020 American Community Survey.

## Data processing

All analyses were conducted on zip-code level data. When data were provided in geographical units other than zipcode, they were converted to zip codes using HUD-USPS crosswalk files, which designate the overlap between zip codes and other geographies.

There is not a one-to-one mapping between zip codes and census units. For example, most zip codes span multiple census tracts, and census tracts often span multiple zip codes. For census-tract data, zip-code level statistics were calculated as the weighted sum (for count variables) or weighted mean (for continuous or binary variables) of their constituent census tracts. Note that for binary variables, this process converts binary variables into continuous ones. For example, census tracts are designated as either urban or rural, but a zip code can consist of a mixture of urban and rural tracts, although most (67%) CA zip codes in our analyses were classified as over 90% urban or rural.

For data provided at the county level, if a zip code spanned more than one county, zip code level metrics were calculated using the weighted mean of the statistic for all relevant counties, based on the % of addresses in a zip code that fall within each county.

##

## Study 2

Our analyses used the following data sources:

**Vaccine site opening and closing; city-wide and zip-code level vaccination rates** We used data provided by the City of Chicago providing vaccination status by zip code as well as vaccine site opening and closing. We did not include in the analyses one dataset designated by USPS as a PO Box (specifically, zip code 60666, representing O’Hare airport). We excluded four remaining residential zip codes in the data because they only included vaccine sites that opened before January 25, 2021—the point in which vaccines first became available to Chicagoans 65 years old and over. This is because prior to January 25 2021, vaccines were only available to a highly limited subset of the population (“Healthcare workers and long-term care facility staff and residents”).^9^ Note that based on the city’s data, some people under the age of 65 received the vaccine before becoming officially eligible.

**Population density, median household income, share of seniors, and share of non-Hispanic white population** We used zip-code level census data for population density (number of residents per square mile), urban/rural status, median family income, and share of seniors, obtained using the “zipcodeR” package. The share of the non-Hispanic white population was taken from the 5-year 2020 American Community Survey.

**References - Supplementary Information**

1. California Department of Public Health. COVID-19 Vaccine Progress Dashboard Data by ZIP Code. Updated January 4, 2021. Accessed January 7, 2021. <https://data.chhs.ca.gov/dataset/covid-19-vaccine-progress-dashboard-data-by-zip-code>

2. GISCorps. Covid-19 Vaccination Provider Locations in the United States. Covid-19 Vaccination Provider Locations in the United States. Published October 25, 2021. Accessed November 26, 2021. <https://coronavirus-resources.esri.com/datasets/GISCorps::covid-19-vaccination-provider-locations-in-the-united-states/about>

3. HHS. Vaccine Hesitancy for COVID-19: County and local estimates. Published 2021. Accessed November 26, 2021. <https://data.cdc.gov/Vaccinations/Vaccine-Hesitancy-for-COVID-19-County-and-local-es/q9mh-h2tw>

4. Voting and Election Science Team. 2020 Precinct-Level Election Results. Harvard Dataverse. Published 2021. Accessed November 26, 2021. <https://dataverse.harvard.edu/dataset.xhtml?persistentId=doi:10.7910/DVN/K7760H&version=27.0>

5. California Statewide Database. 2020 General Election Geographic Data. Published 2020. Accessed October 15, 2021. <https://statewidedatabase.org/d10/g20_geo_conv.html>

6. HUD. HUD USPS Zip Code Crosswalk Files. Published 2021. Accessed November 26, 2021.<https://www.huduser.gov/portal/datasets/usps_crosswalk.html>

7. City of Chicago. COVID-19 Vaccinations by ZIP Code | City of Chicago | Data Portal. Accessed January 24, 2023. <https://data.cityofchicago.org/Health-Human-Services/COVID-19-Vaccinations-by-ZIP-Code/553k-3xzc>

8. City of Chicago. COVID-19 Vaccination Locations | City of Chicago | Data Portal. Chicago. Accessed January 24, 2023. <https://data.cityofchicago.org/Health-Human-Services/COVID-19-Vaccination-Locations/6q3z-9maq>

9. State of Illinois, Vaccination Plan Overview (September 22, 2023). <https://coronavirus.illinois.gov/vaccines/vaccination-plan-overview>

# 
